# Influencing Factors of *Bidens pilosa* L. Hyperaccumulating Cadmium Explored by the Real-Time Uptake of Cd^2+^ Influx around Root Apexes under Different Exogenous Nutrient Ion Levels

**DOI:** 10.3390/toxics11030227

**Published:** 2023-02-27

**Authors:** Siqi Wang, Huiping Dai, Dandan Ji, Shuang Cui, Chengzhi Jiang, Lidia Skuza, Lianzhen Li, Dariusz Grzebelus, Shuhe Wei

**Affiliations:** 1 Academy of Environmental and Chemical Engineering, Shenyang Ligong University, Shenyang 110159, China; 2 Shaanxi Province Key Laboratory of Bio-Resources, Qinling-Bashan Mountains Bioresources Comprehensive Development C.I.C, State Key Laboratory of Biological Resources and Ecological Environment Jointly Built by Qinba Province and Ministry, College of Biological Science & Engineering, Shaanxi University of Technology, Hanzhong 723001, China; 3 Key Laboratory of Pollution Ecology and Environment Engineering, Institute of Applied Ecology, Chinese Academy of Sciences, Shenyang 110016, China; 4 Centre for Molecular Biology and Biotechnology, Institute of Biology, University of Szczecin, 71-415 Szczecin, Poland; 5 College of Environmental Sciences and Engineering, Qingdao University, Qingdao 266071, China; 6 Department of Plant Biology and Biotechnology, Faculty of Biotechnology and Horticulture, University of Agriculture in Krakow, 31-120 Krakow, Poland

**Keywords:** *Bidens pilosa* L., Cd, root tips, influx, chlorophyll, root vigor

## Abstract

Though *Bidens pilosa* L. has been confirmed to be a potential Cd hyperaccumulator, the accumulation mechanism is not yet clear. The dynamic and real-time uptake of Cd^2+^ influx by *B. pilosa* root apexes was determined using non-invasive micro-test technology (NMT), which partly explored the influencing factors of the Cd hyperaccumulation mechanism under the conditions of different exogenous nutrient ions. The results indicated that Cd^2+^ influxes at 300 μm around the root tips decreased under Cd treatments with 16 mM Ca^2+^, 8 mM Mg^2+^, 0.5 mM Fe^2+^, 8 mM SO_4_^2−^ or 18 mM K^+^ compared to single Cd treatments. The Cd treatments with a high concentration of nutrient ions showed an antagonistic effect on Cd^2+^ uptake. However, Cd treatments with 1 mM Ca^2+^, 0.5 mM Mg^2+^, 0.5 mM SO_4_^2−^ or 2 mM K^+^ had no effect on the Cd^2+^ influxes as compared with single Cd treatments. It is worth noting that the Cd treatment with 0.05 mM Fe^2+^ markedly increased Cd^2+^ influxes. The addition of 0.05 mM Fe^2+^ exhibited a synergistic effect on Cd uptake, which could be low concentration Fe^2+^ rarely involved in blocking Cd^2+^ influx and often forming an oxide membrane on the root surface to help the Cd uptake by *B. pilosa*. The results also showed that Cd treatments with high concentration of nutrient ions significantly increased the concentrations of chlorophyll and carotenoid in leaves and the root vigor of *B. pilosa* relative to single Cd treatments. Our research provides novel perspectives with respect to Cd uptake dynamic characteristics by *B. pilosa* roots under different exogenous nutrient ion levels, and shows that the addition of 0.05 mM Fe^2+^ could promote the phytoremediation efficiency for *B. pilosa*.

## 1. Introduction

Cadmium (Cd), a major pollutant of soils, is liable to transportation and accumulation in plants. The morphology and structure of roots are affected by Cd toxicity and this results in elongation limitation, epidermal tissue disturbance and root hair number decrease [1]. However, the roots of hyperaccumulators have a strong capability to uptake and immobilize Cd by compartmentalizing in cell walls and vacuoles, thus alleviating Cd stress [2]. Lan et al., (2018) discovered that the Cd-resistance mechanisms of *Microsorum pteropus* Copel. roots were the regulation of antioxidant enzyme system and the enhancement of energy metabolism [3]. The amount of bioavailable Cd was mainly determined by the soil characteristics, and the Cd bioavailability was influenced by multiple factors in soils, such as: soil texture, pH, CEC, contents of organic matter, clay and nutrient ions, etc., which all affected the Cd bioavailability in soils, and further affected Cd accumulation and distribution in plants [4,5].

*Bidens pilosa* L. belongs to the *Bidens* L. genus of Asteraceae. *B. pilosa*, an annual herb and it is a widespread weedy plant that originated from North America. It is characterized by fast growth and strong adaptability to adverse habitats [6,7]. Wei et al., (2008) also found that the Cd content in *B. pilosa* leaves and stems were 192 and 115 mg/kg, respectively, while the Cd concentration in contaminated soil was 100 mg/kg. The enrichment and translocation factors were all greater than 1.0, and the biomass was not significantly affected, as compared to the controls [8]. Sun et al., (2009) verified that the addition of Cd (≤25 mg/kg) increased the growth of *B. pilosa* by 34.5–104.4%. Cd content in stems and leaves was 103 and 110 mg/kg, respectively, and the bioaccumulation and transfer factors were 3.0 and 7.9, when the Cd concentration was 10 mg/kg in soils [9]. Han et al., (2020) showed that the aqueous extract from the stem of *B. pilosa* increased Cd accumulation capacity of *Solanum nigrum* L. by 44%, as compared to the controls, when the Cd concentration was 1.5 mg/kg soil. They also found that the addition of the aqueous extract improved enzyme activities and the number of microorganisms in soils [10]. Wei et al., (2018) suggested that the antioxidant enzyme activities of *B. pilosa* could up-regulate to cope with Cd stress, for instance, the CAT and POD activities of leaves increased significantly under the 10~40 mg/kg Cd stress relative to the controls, but the enzyme activities decreased under the 60 mg/kg Cd concentration. The height of plant was up to peak under treatment with 40 mg/kg Cd, and the net photosynthetic rate and the biomass of roots and stems were increased after exposure to both 40 mg/kg and 60 mg/kg Cd, but leaf biomass was not impacted significantly by the Cd treatments [11]. Dai et al., (2021) found that no significant differences were observed from chlorophyll a and b in the *B. pilosa* leaves between the controls and treatment with 2.57 mg/kg Cd, and the concentrations of chlorophyll increased with the increase in Cd contents in soils [12].

Non-invasive micro-test technology (NMT) has been applied universally in the detection of the dynamic and real-time uptake of ions by plant roots and it primarily originated from the advantages of dynamic measurements, non-destructive, multi-site scanning and high precision [13]. Wang et al. (2022) discovered that the highest Cd^2+^ influx appeared at 300 μm from the soybean seedling root apex. He also showed that the differences in Cd^2+^ influx to the root tips existed between different soybean cultivars and Cd^2+^ influxes increased significantly after pre-exposure to 5 µM Cd^2+^ compared with controls [14]. Wu et al., (2019) explored the impacts of exogenous Zn on Cd uptake by pakchoi roots by NMT. He also suggested that the net Cd^2+^ influx of pakchoi roots reduced with the increase in Zn levels, and the Cd^2+^ influx at 200 μm from the root quiescent center was inhibited under treatments with 1μM Zn^2+^ as compared to the controls [15]. Sun et al., (2013) explored the spatial characteristics of Cd transport in two ecotypes of *Sedum alfredii* Hance by using NMT and demonstrated that the highest Cd^2+^ influxes were mainly distributed in the root tips and root hairs [16]. Li et al., (2017a) selected three common wetland plant species as materials to explore the Cd uptake mechanisms by plants under NMT application and found that the highest Cd^2+^ influx was located in the meristematic zone (at positions 100 and 200 μm from the root tip of *Typha latifolia* Linn. and *Canna indica* L.) under 10 μM Cd stress and the Cd^2+^ fluxes reversed from efflux to influx at 500 μm of the root apex of *Phragmites australis* Trin. [17].

Numerous studies have concentrated on Cd enrichment by different organs of *B. pilosa* recently. Nevertheless, the quantification and real-time monitoring of Cd uptake by *B. pilosa* roots has been a conundrum up to now, and little research has been completed in support of the Cd^2+^ dynamic uptake. Thus, this experiment aimed to investigate the effects of different exogenous nutrient ions on Cd uptake by *B. pilosa* roots with the application of NMT. These new discovers are quite new. We hypothesized that the Cd^2+^ influx in *B. pilosa* roots exhibited significant differences under Cd treatments with different exogenous nutrient ions.

## 2. Materials and Methods

### 2.1. Plant Culture and Treatment

The experiment was conducted in a greenhouse located in the Shenyang Institute of Applied Ecology of the Chinese Academy of Sciences (123°59′ E and 41°92′ N). The seeds of *B. pilosa* were collected from plots and fields in the experimental station when they reached the maturity phase. The root integrity and activity were crucial to *B. pilosa* as qualified experimental materials in NMT and the sand culture was in favor of root growth and kept the root from rotting. So, sand culture was the main cultivation method in our experimental design. Twenty seeds of *B. pilosa* were sown in pots filled with sterile sand at a depth of 2 cm after prior surface disinfection. The seeds were cultivated in the greenhouse at 24 ± 2 °C at an 8 h/16 h dark/light cycle. Before germination, Hoagland’s solution was poured into each pot with 20 mL every day. After germination, uniform seedlings were screened to ten plants per pot, and intact seedlings of *B. pilosa* were treated with 10 μM Cd^2+^ (as CdCl_2_ × 2.5 H_2_O), KH_2_PO_4_ was replaced with KCl to avoid the formation of Cd phosphate precipitations in solution, and different concentrations of inorganic ions Ca^2+^, Mg^2+^, Fe^2+^, SO_4_^2−^, Na^+^ and K^+^ treatments were used as shown in the Table 1. The pH of the solutions was kept at 5.5 ± 0.2 by the addition of 2 mM MES (2-morpholinoethanesulphonic acid). Meanwhile, the sterile nutrient solutions without Cd^2+^ were used as controls and 20 mL was poured every day. Every treatment was repeated three times and each pot was arranged randomly during the experiments. The plants were harvested after ten days from germination.

### 2.2. Measurements of Cd^2+^ Fluxes by NMT

Firstly, the primary root from the intact plant of *B. pilosa* seedlings was selected and fixed loosely in the measuring chamber. Secondly, the preparation of mother solutions consisting of 0.1 mM Ca(NO_3_)_2_, 0.1 mM KNO_3_, and 1 mM NaCl was completed and the measuring solution (100 μM Cd^2+^) and calibration solutions (50 μM Cd^2+^ and 500 μM Cd^2+^) were obtained on the basis of the mother solutions. Secondly, the roots of *B. pilosa* seedlings were cleaned and soaked in the measuring solution, then the roots were fixed in a culture dish filled with measuring solution. The Cd^2+^ fluxes from the root apex of *B. pilosa* were determined by the scanning ion-selective electrode technique (SIET system BIO-001A; Younger USA, LLC, Lothian, MA, USA). The positive values represent effluxes and negative values represent influxes. Meanwhile, the cadmium ion selective microelectrode was corrected by calibration solutions during testing. The scanning locations from the root apex were 0 μm, 100 μm, 200 μm, 300 μm, 400 μm, 500 μm, 600 μm, 700 μm and 800 μm and the data were processed using Mage Flux [14,18,19].

### 2.3. Measurements of Chlorophyll, Carotenoid and Root Vigor

Briefly, the fresh leaf samples of *B. pilosa* seedling were homogenized in 95% ethanol by using a pre-chilled pestle and mortar, and then the homogenates were centrifuged at 10,000 rpm for 20 min at 4 °C. The supernatant was used to separate chlorophyll a, b and carotenoid by 80% acetone. The concentrations of chlorophyll a, b and carotenoid were determined by a UV-visible near infrared spectrophotometer (UV-3600i Plus) at 665 nm, 649 nm and 470 nm [20,21]. Root vigor is a significant indicator of inorganic ion uptake by roots, and it is characterized by dehydrogenase activity, which is measured by the reduction amount of triphenyl tetrazole chloride (TTC) per unit time [22].

### 2.4. Determination of Biomass, Cd Concentration and Quality Control

The harvested roots and shoots of *B. pilosa* were separated and washed with ultra-pure water. The samples were oven dried at 100 °C for 30 min and then at 80 °C to a constant weight. The biomass was measured by a balance accurate to 0.001 g.

Briefly, the roots of *B. pilosa* seedlings were soaked in 0.02 M EDTA solution to remove any non-specifically bound Cd, and subsequently washed with deionized water, oven-dried for 35 min at 100 °C, then at 50 °C until constant weight, ground to powders and digested with a mixture of concentrated HNO_3_ and HClO_4_ (87:13, *v/v*) [23]. Meanwhile, the inductively coupled plasma optical emission spectrometer (ICP-OES) method was used to determine Cd concentration in all samples (Optima 8000) [24]. The measured contents of Cd in plants of *B. pilosa* seedlings were checked by using standard reference material for plant composition analysis (GBW07604, GSV-3, poplar leaves), and the Cd recovery rate was 91 ± 2% after determination [25].

### 2.5. Data Processing and Statistical Analysis

Microsoft EXCEL 2010 was used to calculate the average and standard deviation (SD) and for preparation of graphs. Data statistical analysis was conducted by SPSS 25.0 and DPS (V 9.01). Data in figures are shown as mean ± standard deviation (*n* = 3). Least significant difference (LSD) tests were used by for evaluating significant differences among Cd^2+^ influxes under different treatments at *p* < 0.05 level with different uppercase or lowercase letters, and a one-way ANOVA was used to compare the means of the physiological index under different treatments at *p* < 0.05 level with different uppercase or lowercase letters [26,27,28].

## 3. Results

### 3.1. Effects of Cd Treatments with Different Nutrient Ions on Cd^2+^ Influxes to the Root of B. pilosa

**Figure 1:**
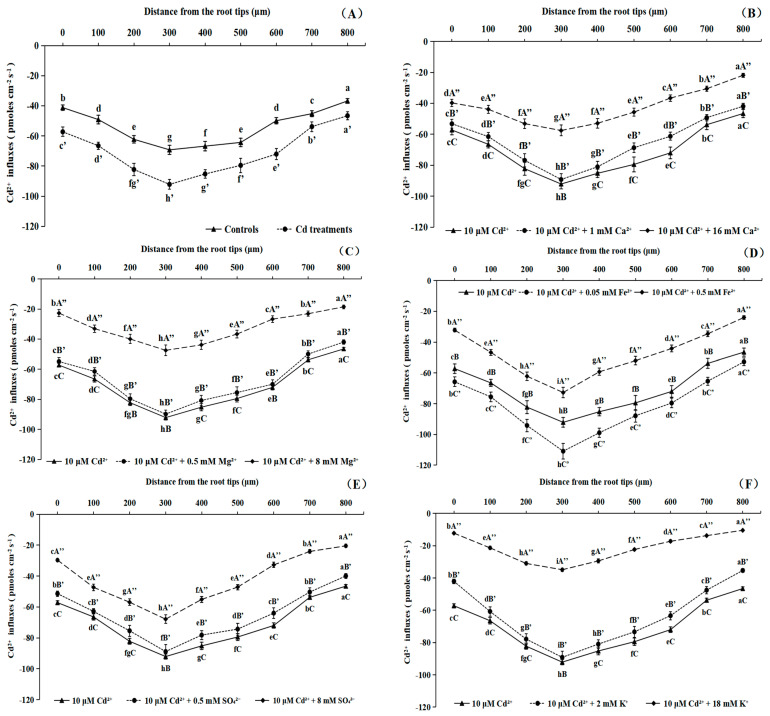
The Cd^2+^ influx at the root surface of *Bidens pilosa* L. from control groups (**A**) and Cd treatment groups with different concentrations of Ca^2+^ (**B**), Mg^2+^ (**C**), Fe^2+^ (**D**), SO_4_^2−^ (**E**) and K^+^ (**F**). Notes: The negative values of Cd^2+^ net flux denote the influx from the measuring solution to the root. The means of Cd^2+^ influx at different distances from the root tips marked with the same lowercase letters (a–h) were not significantly (*p* > 0.05) different, and the means of Cd^2+^ influx under different treatments marked with the same capital letters (A–C) were not significantly (*p* > 0.05) different.

As shown in Figure 1A, the negative values of the net flux denoted the influxes from the solutions to the roots. So, the comparative analyses of Cd^2+^ influxes under different treatments were based on absolute values. Cd^2+^ influxes to the root apexes of Cd-treated plants were higher than that of the controls. The results also showed an obvious spatial distribution of Cd^2+^ influxes, with the highest Cd^2+^ influxes located at 300 μm from the root apex, which could be a typical site for an analysis of the differences in Cd uptake by B. pilosa roots under Cd treatments with different exogenous ions. The influxes gradually decreased at other locations around this site (Figure 1A). Uptake of Cd^2+^ into the root of B. pilosa was inhibited by Cd treatments with 16 mM Ca^2+^ and 8 mM Mg^2+^. However, the Cd treatments with 1 mM Ca^2+^ and 0.5 mM Mg^2+^ had little effect on the net Cd^2+^ influxes at 300 μm from the root tip, as compared to the Cd treatment alone (Figure 1B,C). Cd^2+^ uptake by B. pilosa roots was also reduced by adding 0.5 mM Fe^2+^ and 8 mM SO_4_^2−^. The Cd treatment with 0.05 mM Fe^2+^ significantly increased (*p* < 0.05) the Cd^2+^ influxes from B. pilosa roots (Figure 1D). However, no significant differences (*p* > 0.05) were observed between Cd treatments with 0.5 mM SO_4_^2−^ and Cd stress alone (Figure 1E). Upon the increase in the K^+^ concentration in solutions from 2 mM to 18 mM, the net Cd^2+^ influx of the roots decreased by 62.14% at 300 μm from the root tips. However, there were no significant (*p* > 0.05) differences between Cd treatments with 2 mM K^+^ and the Cd treatments alone (Figure 1F).

### 3.2. Effects of Cd Treatments with Different Nutrient Ions on Biomass and Cd Accumulation of B. pilosa

**Figure 2:**
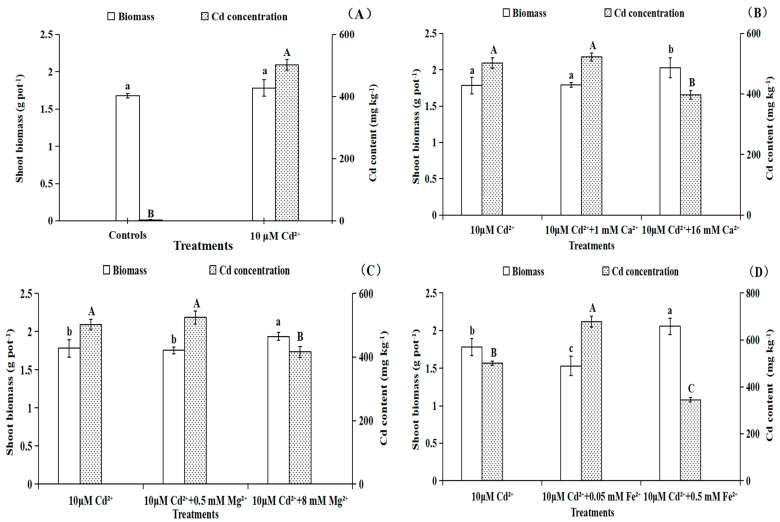

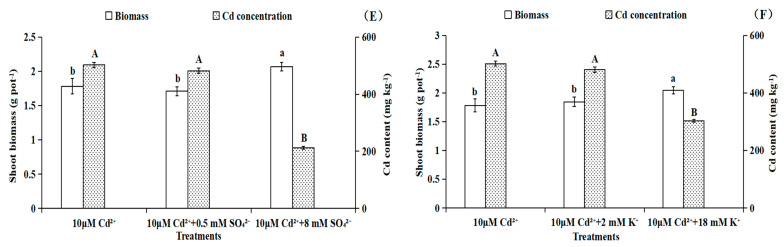
The shoot biomass and Cd contents of *Bidens pilosa* L. from control groups (**A**) and Cd treatment groups with different concentrations of Ca^2+^ (**B**), Mg^2+^ (**C**), Fe^2+^ (**D**), SO_4_^2−^ (**E**) and K^+^ (**F**). Notes: the Cd contents were from the whole seedling of *B. pilosa*, the means of biomass under different treatments marked with the same lowercase letters (a–c) were not significantly (*p* > 0.05) different, and the means of Cd contents under different treatments marked with the same uppercase letters (A–C) were not significantly (*p* > 0.05) different.

As shown from Figure 2A, the shoot biomass of *B. pilosa* did not change significantly (*p* > 0.05) under 10 μM Cd stresses compared with the controls, which indicates that *B. pilosa* conformed to the basic characteristics of a Cd hyperaccumulator. Cd treatments with 16 mM Ca^2+^ and 8 mM Mg^2+^ significantly (*p* < 0.05) promoted the growth of *B. pilosa* as compared to single Cd treatments. However, no significant (*p* > 0.05) changes were observed between Cd treatments with 1 mM Ca^2+^, 0.5 mM Mg^2+^ and Cd treatment alone (Figure 2B,C). The shoot biomass increased significantly (*p* < 0.05) by 15.33% and 15.95% under Cd treatments with 0.5 mM Fe^2+^ and 8 mM SO_4_^2−^, respectively, as compared to the Cd stress alone. However, Cd treatment with 0.05 mM Fe^2+^ decreased the biomass significantly (*p* < 0.05) by 14.20% (Figure 2D,E). A significant (*p* < 0.05) increase in shoot biomass (14.96%) of *B. pilosa* was found for Cd treatments with 18 mM K^+^. However, Cd treatments with 2 mM K^+^ had no effect on biomass relative to the Cd treatment alone (Figure 2F).

The Cd contents of *B. pilosa* plants were 501.95 mg/kg under 10 μM Cd treatments (Figure 2A). The Cd treatments with 16 mM Ca^2+^ and 8 mM Mg^2+^ significantly (*p* < 0.05) reduced the Cd accumulation of *B. pilosa*, as compared to the Cd stress alone. However, no significant (*p* > 0.05) changes were observed between Cd treatments with 1 mM Ca^2+^, 0.5 mM Mg^2+^ and Cd treatment alone (Figure 2B,C). Cd contents of *B. pilosa* were significantly (*p* < 0.05) decreased by 31.19% and 57.77% under Cd treatments with 0.5 mM Fe^2+^ and 8 mM SO_4_^2−^, respectively, while the Cd contents increased by 35.06% when the plants were Cd-treated with the addition of 0.05 mM Fe^2+^, as compared to Cd treatment alone. However, Cd contents did not change significantly (*p* > 0.05) under Cd treatments with 0.5 mM SO_4_^2−^ relative to the Cd stress alone (Figure 2D,E). A significant (*p* < 0.05) reduction in Cd contents (39.63%) was found under Cd treatments with 18 mM K^+^. Furthermore, Cd treatments with 2 mM K^+^ had no effect on Cd content (Figure 2F).

### 3.3. Impacts of Cd Treatments with Different Nutrient Ions on Chlorophyll a and b of B. pilosa

**Figure 3:**
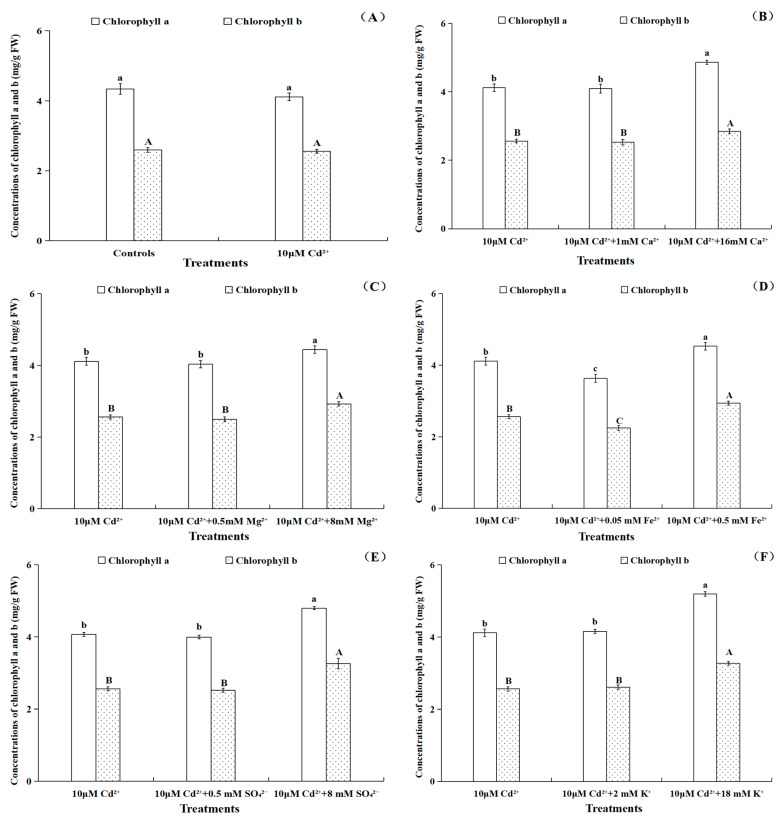
The concentrations of chlorophyll a and b of *Bidens pilosa* L. from control groups (**A**) and Cd treatment groups with different concentrations of Ca^2+^ (**B**), Mg^2+^ (**C**), Fe^2+^ (**D**), SO_4_^2−^ (**E**) and K^+^ (**F**). Notes: the means of chlorophyll a concentrations under different treatments marked with the same lowercase letters (a–c) were not significantly (*p* > 0.05) different, and the means of chlorophyll b concentrations under different treatments marked with the same uppercase letters (A–C) were not significantly (*p* > 0.05) different.

As shown from Figure 3A, the chlorophyll a and b of *B. pilosa* did not have significant (*p* > 0.05) variations with 10 μM Cd treatments, which indicated that *B. pilosa* exhibited remarkable tolerance as a Cd hyperaccumulator. The concentrations of chlorophyll a and b from *B. pilosa* leaves under Cd treatments with different nutrient ions showed the same change trends (Figure 3). There were no significant (*p* > 0.05) differences between the Cd treatments with 1 mM Ca^2+^, 0.5 mM Mg^2+^ and single Cd treatments. Obviously, the chlorophyll a and b concentrations under Cd treatments with 16 mM Ca^2+^ and 8 mM Mg^2+^ were all higher than that of Cd stress alone (Figure 3B,C). The concentrations of chlorophyll a and b significantly (*p* < 0.05) increased by 10.04%, 14.96% and 17.77%, 27.05% under Cd treatments with 0.5 mM Fe^2+^ and 8 mM SO_4_^2−^, respectively, as compared to single Cd treatments. The concentrations of chlorophyll a and b decreased by 11.82% and 12.61% under Cd treatments with 0.05 mM Fe^2+^, respectively, as compared with single Cd treatments. However, the concentrations of chlorophyll a and b showed no significant (*p* > 0.05) differences when the plants were Cd-treated with 0.5 mM SO_4_^2−^ as compared with the Cd stress alone (Figure 3D,E). Furthermore, the concentrations of chlorophyll a and b under Cd treatment with 18 mM K^+^ were significantly (*p* < 0.05) higher (26.15% and 27.57%) than that of Cd treatment alone. However, Cd treatments with 2 mM K^+^ had no effect on the concentrations of chlorophyll a and b (Figure 3F).

### 3.4. Effects of Cd Treatments with Different Nutrient Ions on Carotenoid and Root Vigor of B. pilosa

**Figure 4:**
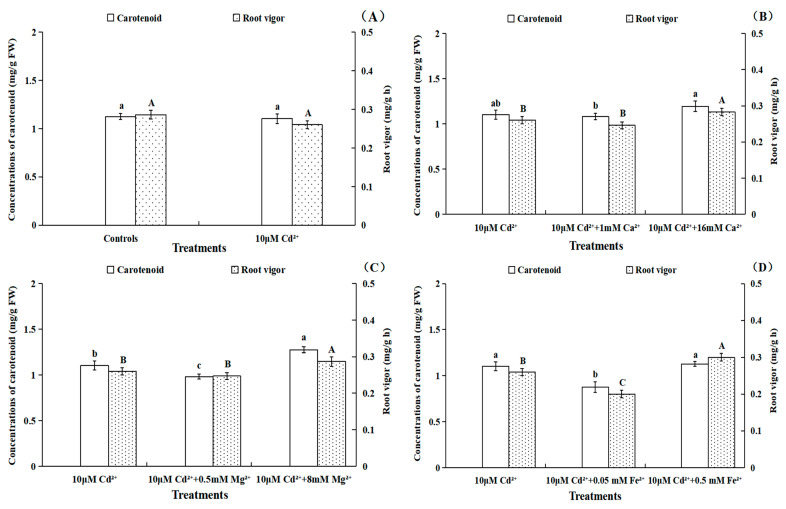

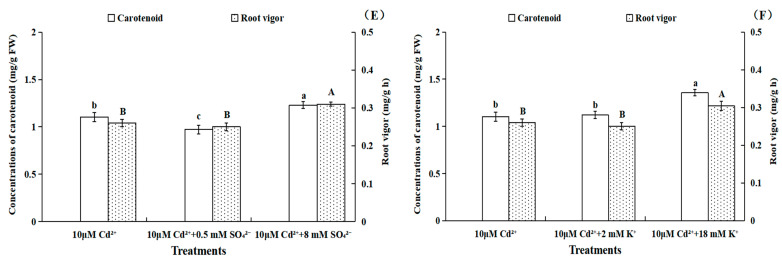
The concentrations of carotenoid and root vigor of *Bidens pilosa* L. from control groups (**A**) and Cd treatment groups with different concentrations of Ca^2+^ (**B**), Mg^2+^ (**C**), Fe^2+^ (**D**), SO_4_^2−^ (**E**) and K^+^ (**F**). under Cd treatments with different nutrient ion levels. Notes: the means of carotenoid concentrations under different treatments marked with the same lowercase letters (a–c) were not significantly (*p* > 0.05) different, and the means of root vigor under different treatments marked with the same uppercase letters (A–C) were not significantly (*p* > 0.05) different.

There were no significant (*p* > 0.05) changes in carotenoid concentrations and root vigor under 10 μM Cd treatments compared with controls; this was mainly determined by the tolerance of *B. pilosa* seedlings to Cd (Figure 4A). No significant (*p* > 0.05) variations of carotenoid concentrations were observed under Cd treatments with 1 mM and 16 mM Ca^2+^ compared with single Cd treatments, but the Cd treatments with 8 mM Mg^2+^ exhibited a significant (*p* < 0.05) increase (15.44%) in *B. pilosa* seedling leaves (Figure 4B,C). The Cd treatments with 0.05 mM Fe^2+^ significantly (*p* < 0.05) decreased the carotenoid concentrations (20.62%) compared to Cd treatments alone, while no significant (*p* > 0.05) changes were observed between Cd treatments with 0.5 mM Fe^2+^ and the Cd treatment alone (Figure 4D). Furthermore, the carotenoid concentrations with Cd treatment with 8 mM SO_4_^2−^ were significantly (*p* < 0.05) higher (11.44%) than that of single Cd treatments (Figure 4E). The concentrations of carotenoid increased by 23.08% under Cd treatments with 18 mM K^+^, as compared with single Cd treatments (Figure 4F).

The Cd treatments with 16 mM Ca^2+^ and 8 mM Mg^2+^ significantly (*p* < 0.05) increased the root vigor of *B. pilosa* compared to Cd treatment alone, while no significant (*p* > 0.05) changes were observed between Cd treatments with 1 mM Ca^2+^, 0.5 mM Mg^2+^ and the Cd treatment alone (Figure 4B,C). A significant (*p* < 0.05) decrease (23.08%) in root vigor was found in the Cd treatments with 0.05 mM Fe^2+^ as compared to Cd treatments alone. The root vigor was significantly (*p* < 0.05) increased by 15.38% and 20.50% under Cd treatment with 0.5 mM Fe^2+^ and 8 mM SO_4_^2−^, respectively. However, Cd treatments with 0.5 mM SO_4_^2−^ had no effect on root vigor relative to the Cd treatment alone (Figure 4D,E). Furthermore, the root vigor under Cd treatment with 18 mM K^+^ was significantly (*p* < 0.05) higher (16.65%) than that of Cd treatment alone. However, Cd treatments with 2 mM K^+^ had no effect on root vigor relative to Cd stress alone (Figure 4F).

## 4. Discussion

### 4.1. The Dynamic Uptake of Cd^2+^ by Accumulator and Hyperaccumulator Roots

Li et al. (2017b) suggested that the distribution of Cd^2+^ influxes had obvious spatial organization around *Sedum plumbizincicola* seedling root tips, and the influx of Cd^2+^ was significantly higher in the meristematic zone. The Cd^2+^ influx rate was highest about 300 μm from the root tip, and steadily decreased in both directions from this location. In addition, the Cd^2+^ flux of 50 μM Cd^2+^ irradiation group was significantly increased. The addition of Cd^2+^ could regulate and induce transporters that mediate the Cd^2+^ influx in the plasma membrane of *S. plumbizincicola* [29]. The views above were basically consistent with our research results, and the highest Cd^2+^ influxes were located at 300 μm from the root tips of *B. pilosa*. Cd pretreatments could promote Cd^2+^ influx compared with controls. Li et al. (2012) studied the dynamic changes of halophyte *Suaeda salsa* root Cd^2+^ uptake by kinetic experiments and discovered that the net Cd^2+^ flux at the maximum flux location (approximately 150 mm from the root tip). Cd uptake was initially very fast, with a net inflow of Cd^2+^ of about 70 pmol cm^−2^ s^−1^, and then rapidly decreased, reaching a steady-state value about 5 min after the addition [30]. By contrast, the Cd stress concentration in our studies was 10 μM and the net Cd^2+^ influx at 300 μm from the root tip of *B. pilosa* was −92.12 pmol cm^−2^ s^−1^ under medium–low stress.

### 4.2. Impacts of Different Nutrient Ions on Cd^2+^ Fluxes of Roots

He et al., (2015) suggested that the Cd uptake by poplar roots was mediated by Ca, and the peak value of net Cd^2+^ influxes into roots was observed under 0.1 mM Ca^2+^ treatments [31]. Li et al., (2017b) found the Cd^2+^ uptake by *Sedum plumbizincicola* was reduced by 50% when the concentration of K^+^ increased to 10 mM. The net Cd^2+^ flux into the root of *Sedum plumbizincicola* changed from an influx to an efflux when the concentration of Ca^2+^ was up to 1.0 mM in measuring solutions, and the net Cd^2+^ influx decreased slightly by 4% at 300 μm from the root apex under treatment with 1.0 mM Mg^2+^ in solutions [29], Lu et al., (2010) verified that the Cd^2+^ influxes of *Sedum alfredii* roots decreased significantly when Ca^2+^ in nutrient solutions was elevated from 2.0 to 32.0 mM [32]. Perfus-Barbeoch et al. (2002) showed that Cd^2+^ affected guard cell regulation by entering the cytosol of *Arabidopsis thaliana* L. through Ca^2+^ channels [33]. Our results indicated that Cd^2+^ influxes at 300 μm from the root tips decreased by 37.55%, 48.46% and 62.14% under Cd treatments with 16 mM Ca^2+^, 8 mM Mg^2+^ or 18 mM K^+^. The studies listed above were basically consistent with the results of our studies. Rabêlo et al. (2017) studied the influence of S on Cd uptake by Massai grass roots and discovered that the Cd treatments with 1.9 mM SO_4_^2−^ inhibited the symplastic Cd^2+^ influx and improved the apoplastic Cd^2+^ influx in root compared with the Cd treatment alone [34]. Zhang et al., (2020) researched the underlying mechanisms of Cd uptake by the root tips of *Vicia sativa* with application of NMT and suggested that the Cd uptake was closely related to Fe uptake under Cd treatments with different Fe levels [35]. The views above basically agreed with our research, and the Cd^2+^ influxes at 300 μm from the root tips of *B. pilosa* decreased by 26.29% and 21.04%, respectively, under Cd treatments with 8 mM SO_4_^2−^ and 0.5 mM Fe^2+^ compared to the Cd stress alone. The Cd treatments with a high concentration of nutrient ions showed an antagonistic effect on Cd^2+^ uptake. It might be that these ions at a high concentration could take up the Cd^2+^ channels and compete for the binding sites in *B. pilosa* roots.

### 4.3. Effects of Different Nutrient Ions on Biomass, Cd Accumulation and Physicochemical Characteristics of Accumulator and Hyperaccumulator

Li et al., (2017b) found that the presence of 10 mM Ca^2+^ and Mg^2+^ significantly inhibited Cd^2+^ uptake by *Sedum plumbizincicola*, and the higher Ca^2+^ and Mg^2+^ concentrations induced lower Cd accumulation, for instance, the Cd contents in the plants reduced from 1121.8 μg/g to 562.5 μg/g when the concentration of Ca^2+^ shifted from 0.1 mM to 10 mM in solutions [29]. Hakeem et al., (2022) suggested that the root and shoot biomass of *Fagopyrum esculentum* was enhanced by 7.57% and 11.11%, respectively, under 200 mgL^−1^ Cd^2+^ treatments with 300 mgL^−1^ Ca^2+^ when compared to the controls, and the exogenous Ca^2+^ significantly promoted Cd retention in roots and alleviated the Cd-induced oxidative damage [36]. Liu et al. (2020) discovered that 25 μM Cd treatment with 0.5 mM Ca significantly increased the Cd contents in the roots of *Sedum alfredii*, but the Cd contents in shoots decreased under Cd treatment with 8 mM Ca [37]. Lu et al., (2010) showed that the shoot biomass of *Sedum alfredii* increased under Cd treatments with 8.0 mM Ca^2+^ compared with controls, but the biomass decreased obviously when the Ca^2+^ concentration was greater than 8.0 mM [32]. Our results suggested that Cd treatment with 16 mM Ca^2+^ induced the shoot biomass of *B. pilosa* to rise relative to the Cd stress alone, and no significant changes were observed under Cd treatments with 1 mM Ca^2+^. It was possible that the biomass would be promoted as Ca^2+^ concentration went up. Tian et al., (2011) explored the effects of Ca application on the antioxidant systems of *Sedum alfredii* H. roots under Cd-induced oxidative stress and found that the addition of exogenous Ca obviously improved root elongation and decreased the Cd contents in the root apex. The activities of superoxide dismutase (SOD) and catalase (CAT) reduced and the biosynthesis of glutathione (GSH) was promoted in the roots of *S. alfredii* under 400 μM Cd treatments with 6.0 mM Ca compared with single Cd stress [38]. Rabêlo et al., (2018) evaluated the S influence on alleviating Cd damage in Massai grass and suggested that the sufficient S supply (1.9 mmolL^−1^) was conducive to the uptake of Cd and the adequate S supply promoted the root length and surface, Cd translocation factor and the contents of other nutrients [39].

## 5. Conclusions

In summary, the Cd^2+^ influxes of *B. pilosa* root under different nutrient ions were measured by NMT, which indicated that Cd^2+^ influxes at 300 μm around the root apexes decreased under Cd treatments with 16 mM Ca^2+^, 8 mM Mg^2+^, 0.5 mM Fe^2+^, 8 mM SO_4_^2−^ or 18 mM K^+^ compared with single Cd treatments. The Cd treatments with a high concentration of nutrient ions showed an antagonistic effect on Cd^2+^ uptake. However, the Cd treatments with 1 mM Ca^2+^, 0.5 mM Mg^2+^, 0.5 mM SO_4_^2−^ or 2 mM K^+^ had little effect on the net Cd^2+^ influxes compared with the Cd treatment alone. Importantly, Cd treatment with 0.05 mM Fe^2+^ promoted Cd^2+^ uptake by the *B. pilosa* roots. The addition of 0.05 mM Fe^2+^ exhibited a synergistic effect on Cd uptake, which could be low concentration Fe^2+^ rarely involved in blocking Cd^2+^ influx and formed oxide membrane on root surface to help the Cd uptake by *B. pilosa*. The results also showed that the Cd treatments with a high concentration of nutrient ions significantly increased the concentrations of chlorophyll and carotenoid in leaves and the root vigor of *B. pilosa* relative to the Cd treatments alone. Our research has provided novel perspectives with respect to Cd uptake dynamic characteristics by *B. pilosa* roots under different exogenous nutrient ion levels, and the future research could focus on the effects of 0.05 mM Fe addition on the phytoremediation efficiency for *B. pilosa* by pot and field experiments.

## Figures and Tables

**Table 1:** Treatment design of additives to Hoagland solution in pots with *Bidens pilosa* L.

Treatments	Treatment Details
Controls	Hoagland solution
Cd treatments alone	Hoagland solution (10 μM Cd^2+^)
Cd treatments with Ca^2+^	Hoagland solution (10 μM Cd^2+^ + 1 mM Ca^2+^, other ingredients unchanged)
	Hoagland solution (10 μM Cd^2+^ + 16 mM Ca^2+^, other ingredients unchanged)
Cd treatments with Mg^2+^	Hoagland solution (10 μM Cd^2+^ + 0.5 mM Mg^2+^, other ingredients unchanged)
	Hoagland solution (10 μM Cd^2+^ + 8 mM Mg^2+^, other ingredients unchanged)
Cd treatments with Fe^2+^	Hoagland solution (10 μM Cd^2+^ + 0.05 mM Fe^2+^, other ingredients unchanged)
	Hoagland solution (10 μM Cd^2+^ + 0.5 mM Fe^2+^, other ingredients unchanged)
Cd treatments with SO_4_^2−^	Hoagland solution (10 μM Cd^2+^ + 0.5 mM SO_4_^2−^, other ingredients unchanged)
	Hoagland solution (10 μM Cd^2+^ + 8 mM SO_4_^2−^, other ingredients unchanged)
Cd treatments with K^+^	Hoagland solution (10 μM Cd^2+^ + 2 mM K^+^, other ingredients unchanged)
	Hoagland solution (10 μM Cd^2+^ + 18 mM K^+^, other ingredients unchanged)

## Data Availability

The datasets used or analyzed during the current study are available from the corresponding author on reasonable request.
